# Mapping the content of mothers’ knowledge, attitude and practice towards universal newborn hearing screening for development of a KAP survey tool

**DOI:** 10.1371/journal.pone.0210764

**Published:** 2019-02-20

**Authors:** Christine Graham, Janet Seeley, Ayanda Gina, Yougan Saman

**Affiliations:** 1 Nelson Mandela School of Clinical Medicine, University of KwaZulu Natal, Durban, South Africa; 2 Department of Global Health and Development, London School of Hygiene and Tropical Medicine, London, United Kingdom; 3 Department of Audiology, University of KwaZulu Natal, Durban, South Africa; 4 Ear Nose and Throat Department, University Hospitals of Leicester, Leicester, United Kingdom; University of Kentucky, UNITED STATES

## Abstract

Understanding mother’s knowledge, attitude and practice (KAP) of permanent childhood hearing impairment (PCHI) is essential for the success of universal newborn hearing screening (UNHS) as poor compliance and follow-up remains a global challenge. To determine content area for a questionnaire that measures PCHI-related KAP in rural mothers, we trained moderators who interviewed 145 pregnant women (17 groups) from 5 ante-natal clinics. Interviews were recorded, transcribed, summarised and analysed using thematic framework analysis. Four knowledge themes were identified: 1) PCHI was perceived as the malfunction of hearing leading to disability; 2) a poorly-responsive/communicative child may have PCHI; 3) lifestyle, hereditary and environmental factors are significant causes of PCHI; 4) medical management of PCHI was doubted, with some advocating birth and ancestral rituals. Two themes were identified for attitude: 1) beliefs that PCHI was emotionalised due to the negative lifelong impact on the child and family; 2) UNHS processes were favourable though some preferred other belief systems. Three themes were identified for practice: 1) doctors were the first choice followed by traditional healers; 2) willingness to continue follow-up although challenges exist; 3) minimal family support during consultation. The contextualised KAP of women regarding UNHS processes and PCHI provided content area for the design of a KAP tool.

## Introduction

Permanent childhood hearing impairment (PCHI) is a significant cause of disability and can have an enduring impact on cognitive, emotional and social development particularly with regards to the functional limitations of speech and language acquisition [1]. Hearing loss may be present at birth and can result from environmental and prenatal factors, congenital infections and genetic causes [2]. Hypoxia, hyperbilirubinemia, meningitis, chronic otitis media, mumps, measles, cytomegalovirus, trauma, ototoxic drugs and head injury are causes of neonatal and childhood hearing loss [3].

The reported prevalence of disabling hearing loss varies globally as it depends on context. In a well- resourced country such as the United States the estimates are 1.83/1000 newborns rising to 2.7/1000 before the age of five years and 3.5/1000 during adolescence [4]. In sub-Saharan Africa disabling hearing loss is estimated at 1.9% which translates to 19 children per 1000 [5].

Universal newborn hearing screening (UNHS) has become the standard of care in many countries. In the United States and the United Kingdom there are established programmes where almost all babies are screened shortly after birth. Early hearing detection and intervention (EHDI) attempts to lessens the impact on the family and the child as UNHS services aim to be accessible, coordinated and culturally sensitive to support the child, family and community [6]. However, diagnostic follow-up and effective compliance for intervention may be more difficult to achieve as it requires the pragmatic partnership between the health service and families and even in well-resourced settings this remains a challenge [7]. The cost-effectiveness of such programmes however is increasingly becoming evident as we mitigate against the effects of a lifetime of disability. However, in poorly resourced settings, with many competing health priorities, there must be good evidence that the programme can deliver good compliance before policy makers are willing to invest.

For a programme to be successful and cost-effective it is essential that the community, defined as users of early hearing detection services, participates at the level of screening, follow-up as well as for diagnostic procedures and interventions [8]. This will require multiple, time-consuming visits often at the patient’s own expense and where access to healthcare may be challenging. Hence, there is a need to understand the effect of socio-cultural processes on hearing loss and disability in local communities, particularly mothers, so that we can tailor health promotion materials that target their needs [9]. The majority of the literature assesses knowledge and attitude in relation to the experience of NHS that are already in operation [10–15] with relatively few studies undertaken across the world to determine maternal views on HL and the attitudes towards the screening prior to the commencement of a programme.

Accordingly, in this study we determined content area regarding KAP towards UNHSP process and childhood hearing loss (CHL) of expectant women at Amajuba district as part of the process of developing a KAP survey tool. In this study, knowledge is understood as perspectives; attitude as positive and negative inclinations and practice as the action taken in encountering issues of HL or UNHSP process. Using group interviews we initiated a purposeful exchange with women, regarding UNHSP content, to provide the framework in which to understand KAP. The KAP constructed by women is a representation of concepts and meaning that is powerful and thorough about CHL and UNHS. This representation can underline themes for developing a KAP survey tool that will be connecting within participants cultural framework.

## Methods

### Study area

The study was conducted in ante-natal clinics in a rural community in KwaZulu-Natal, South Africa where there are no newborn hearing services.

### Study design

The study undertook a qualitative phenomenological approach and used group interviews for data collection. Although the approach is susceptible to researcher and respondent bias, we minimised the potential impact of these biases from the questions, the participants and the facilitators [16]. Facilitators were trained prior to interviews and guided by the interview protocol. This allowed them to present the questions considerately, enabling the participants to disclose their true beliefs, opinions and feelings without distortions [17]. We pursued this approach because of the focus on context and the quest to understand the phenomena studied in a naturalistic setting [17].

### Participants

Being pregnant was a requirement for participation in the study. Although a qualitative approach rarely uses a systematic approach in sampling, this study conducted a simple random sampling so that all mothers could have an equal chance to participate in the study [17]. Recruitment was done through a meticulous process of first obtaining a number of pregnant women registered to attend the clinic on the day. Since the list was numbered, we wrote each number separately in a piece of paper and tossed in a box and picked ten numbers randomly. We then identified the names from the registered list to obtain participants for group interviews. After the selection, we approached the women and invited them to participate in the group interviews. Most of the selected participants in all sites agreed to participate, except for a few who were either not feeling well and others who failed to turn up to the agreed venue. A total of 145 pregnant mothers at 5 clinics (36 –Nellies Farm; 33 –Osizweni 2; 34 Rosary; 22 –Lulama and 20 –Madadeni 5) were recruited for the study.

### The group interviews

Group interview (GI) was the research method for data collection. It is a method characterised by an amalgamation of group interaction and the researchers selected topic. To provide structure, the interviews were guided by semi-structured, open-ended questions that were developed by a thorough literature search conducted in English about CHL, UNHS and KAP studies. It can thus be viewed as group interaction guided by a researcher on a topic [18,19]. We chose GIs because they substantiate the ability to capture social significance of a phenomena in a more effective way than individual interviews. In this case GIs will shed light about women KAP of CHL and newborn hearing screening (NHS) to enable us identify themes for the development of a KAP tool.

GI’s were conducted from April–June 2016, whereby each GI was comprised of five to ten participants and lasted between 45 minutes to 1 hour. A total of 17 GIs were conducted at the five sites: Nellies Farm clinic—4; Osizweni 2 clinic– 4; Rosary clinic– 4; Lulama clinic– 3; and Madadeni 5 clinic– 2. Since the Zulu language is the mother-tongue of the majority of the population, the interview questions guide was also translated in Zulu by a professional from linguistic department of a University. Thereafter, these Zulu questions were shared with health professionals at Newcastle hospital for clarification about the local dialect as this would provide ease and comfort. The GIs were conducted by 5 facilitators recruited locally whose criteria included interpersonal skills, computer literacy as well as fluency in English and Zulu. Local facilitators provided a space for a degree of similarity, based on living in the same locality and speaking the same language as the participants. A day’s training was provided to the facilitators prior the GIs, then they were given an interview protocol for guidance throughout the interviews. The protocol allowed for a standard to be maintained from one group to another and from one site to another [17]. Since the study involved minimal risk due to its non-invasive nature, at the beginning of each interview, a prepared information sheet was read out to the participants and then verbal consent was requested. It was a process informed consent whereby participants were reminded throughout the interview that they had a right to withdraw if they felt uncomfortable [18]. Then personal introductions of the group and demography was collected.

The objective of the interviews was to explore several issues about HL and the UNHS process as identified from the literature comprising the following: maternal views, opinions, perceptions in relation to causes, treatment, detection; their attitudes regarding these issues at personal, family and community level; and maternal routine practices in terms of seeking treatment and assistance networks in the health context. To strengthen the quality of the study we used Lincoln and Guba’s (1985) ‘trustworthiness’ framework that encompasses the criteria of credibility which refers to the truth of the data; dependability referring to the stability of the data, and authenticity refers to the researcher demonstrating faithfully the realities of the study group [18]. Therefore, to establish credibility and authenticity all interviews were audio recorded, transcribed and translated verbatim. In addition, the researcher or the facilitator made observations of each GI and took notes on body language, moods and attitudes as well the overall environment. In terms of dependability, each response which was not clear was taken back to the participant to clarify what they meant. Then an independent reviewer was given the transcripts and the audio to verify the quality of the data. Hence, information about KAP of CHL and UNHS process was described in the words of the expectant mothers and according to the meanings given from their own community. Generally, GIs elicited a wide range of information about women’s ideas and feelings regarding HL and UNHS process, whilst shedding light on the diverse perspective of mothers between and within groups [19].

### Data analysis

Thematic analysis was used to analyse the data. The coding of the transcripts was done manually using a “Microsoft Excel 2016”, a spreadsheet programme which enabled us to build a pattern of participant’s descriptions of ideas and significant statements by focusing on their meanings towards CHL and the UNHS process. It should be noted that the coding was a combination of emerging and predetermined codes, as the questions came from the content of CHL and NHS. Further, the coding allowed us to generate categories that eventually informed us about the emerging themes. Then an inductive process (which is ‘working back and forth between themes’ and transcripts), was used to determine an inclusive set of themes [17]. Thereafter, a deductive process was employed by thoroughly examining the transcripts to verify that all evidence collected was included in the identified themes. To ensure rigor through credibility and dependability of ‘trustworthiness’, GI data within and between study sites was triangulated against the themes and the descriptions in which mothers shared their diverse expressions about CHL and the UNHS process were contrasted and reinforced with extracts from the transcripts. To acquire consistency in all processes from the transcripts, coding and thematic analysis was shared amongst all the authors for verification.

Each transcript was labelled by the first letter of the clinic followed by the group interview number (Nellies Farm–N1, N2, N3, N4; Osizweni 2 –O1, O2, O3, O4; Rosary–R1, R2, R3, R4; Lulama–L1, L2, L3; and Madadeni 5 clinic–M1, M2). The results are presented by “a few, some, several GIs, etc.” to give a broader sense of the weight of the participant’s reality and putting emphasis of the existence of the multiple realities about their understanding of CHL and NHS in their everyday lives. Participants were also identified by a number indicating the site, interview label and age in years. We used study site identification as we assumed that interactions of the diversity of socio-cultural life gives meaning and transmits knowledge of this phenomena within the wider community [20].

### Ethical considerations

The study obtained ethical approval from the Biomedical Research Ethics Committee (BREC)–No. BFC261/16 (sub-study of BFC421/15) at the University of KwaZulu-Natal. Informed consent was obtained from the participants and they were guaranteed confidentiality within possible bounds. Participants had the right to refuse participation as the study was voluntary.

## Findings

The study group comprised of women aged between eighteen and forty years as shown in Table 1 below. It is important to note that there was a large group of single women in the study sample which is representative of the general status in South Africa [21].

**Table 1:** Characteristics of participants.

**Clinics**	**Distribution in GI’s—*N*(Age range)**						Total *N*
	1	2		3	4		
Nellies Farm	8(18–36)	9(18–29)		9(18–36)	10(18–31)		36
Osizweni 2	8(18–36)	10(19–36)		8(22–40)	7(22–35)		33
Rosary	9(18–35)	7(21–31)		10(18–34)	8(18–34)		34
Lulama	5(22–28)	7(24–28)		10(23–31)	-		22
Madadeni 5	10(18–34)	10(18–34)		-	-		20
**Clinics**	**Distribution in Age range–*N(%)***						
	**18–20**		**21–30**			**31–40**	
Nellies Farm (*N* = 36)	16(44)		14(39)			6(17)	
Osizweni 2 (*N* = 33)	7(21)		18(55)			8(24)	
Rosary (*N* = 34)	11(32)		16(47)			7(21)	
Lulama (*N* = 22)	0		21(95)			1(5)	
Madadeni 5 (*N* = 20)	3(15)		12(60)			5(25)	
Total (*N* = 145)	37(26)		81(56)			27(19)	
**Marital Status** (*N* = 145)							
Married	3(2)		11(8)			5(3)	
Single	32(22)		67(46)			20(14)	
Living with a partner	2(1)		3(2)			2(1)	
**Education** (*N* = 145)							
No school	0		1			2(1)	
Primary	16(11)		25(7)			8(6)	
High school	20(14)		49(34)			16(11)	
Higher education	0		6(4)			2(1)	
**Employment** (*N* = 145)							
Employed	2(1)		17(12)			8(6)	
Unemployed	22(15)		55(12)			19(13)	
Students	13(9)		9(6)			0	

*N* = number of participants

The descriptive accounts of women’s knowledge, attitude and practice in the study identified nine themes. The broad phenomena of knowledge, attitude and practice are presented below in a more integrated assessment of the interviews, with the identified themes followed by contextual illustrations. These illustrations identified women aged 18–20 years as younger, those aged 21–30 years as middle-aged and age 31–40 years as older women. The use of age groups simply shows the age dynamics of the study group.

### Knowledge

In exploring knowledge, four themes emerged from the group interviews comprising the perception of deafness, causes of deafness, indication of deafness and detection/treatment.

#### Perception of deafness

The perception of deafness echoed women’s description of deafness or being deaf. Most women, in almost all the GIs (16/17), perceived deafness as the malfunction of the sense of hearing leading to a dysfunction in the child. Some middle-aged and younger women (9%) described deafness as the defects of the ear. They said:

“I think it is a nerve in the ear that is not right” (O1.3, 27 years)

“I think the problem is with the eardrums” (M1.7, 24 years)

“Sound waves do not enter in the ear and the vibrations are not good, it is a blockage in an ear” (O3.2, 20 years)

On the other hand, some participants (43%) across groups considered HL as simply a hearing problem whereby the following older women said:

“It is when you cannot hear the sounds around you” (N4.1, 31 years)

“It is the difficulty in hearing” (M1.3, 33 years)

Women in about half of the GIs (8/17) made suggestions for what should be done if a baby was born with HL. Several middle-aged women (15%) stated the need to get help urgently as the dysfunction of the child may provide an array of challenges in family, school and community settings, verbalised in the following manner:

“The child will have problems at home with other children and when s/he start schooling. The challenges will be hearing others” (M1.5, 25 years)

“Babies born with hearing loss needs to get special treatment before it gets worse. If delayed it can damage baby’s eyes and the baby will be incapacitated.” (N2.2, 29 years)

They suggested that the assistance required from the government or professional agencies as follows:

“The government need to do something like awareness about the problem of hearing loss …as it may save those children” (R3.7, 22 years)

“There is a need for some professional help so that they counsel us and tells us which steps to take so that the babies can get better” (O4.3, 23 years)

Nevertheless, in very few GIs (4/17), some middle-aged women (6%), declared knowing nothing more about deafness than seeing people that are deaf. Further, the perception of a baby being born with a HL was incomprehensible and this was supported by some older women (7%) as well. The articulated statements include:

“I have never heard of a baby being born deaf” (L1.3, 22 years)

“It cannot be, the baby is still young for hearing anything” (L2.5, 27 years)

“There is nothing to think about. It is God’s will” (L3.3, 34 years)

Women’s diverse perspectives of deafness were further described in terms of the causes of HL.

#### Causes of deafness

The descriptions given by participants about the causes of deafness led to the identification of this theme. In the context of newborn hearing loss (NHL), over half of the GIs (9/17) acknowledged that pregnant mothers’ lifestyle behaviours and hereditary factors were the main causes of hearing loss. Mostly, middle-aged women (19%) expressed that pregnant mothers that were exposed to smoking, alcohol and drug consumption as well as poor diet were more likely to have newborns with HL:

“When the pregnant mother uses drugs or consumes alcohol that will affect the baby and become deaf” (N1.2, 21 years)

“I think it may be caused by pregnant mother taking too much alcohol. I have a relative who was doing that when pregnant and her child was born with ears that were always discharging” (M1.5, 25 years)

“Those babies whom their mothers did not get healthy food while pregnant” (N4.2, 26 years)

Several women (10%) in just over half of the GIs (12/17) across all ages mentioned genetics as a probable cause of NHL. These women said:

“It is a hereditary disease, for example, when one member of the family has a hearing problem, then a child might be affected” (R1.7, 19 years)

“It can be hereditary, as there are some conditions which are passed on to children” (L3.7, 28 years)

“Maybe it is genetic, a condition that can be passed on from one generation to another” (M2.1, 33 years)

In over half of the GIs (9/17), women mentioned late attendance at the clinic, non-attendance and non-adherence to health professional advice can lead to NHL. These views were shared by young and middle-aged women (11%) in this way:

“Those babies whom their mother started the clinic late during their pregnancy” (N3.6, 28 years)

“When the mothers are used to not coming to the clinic for immunization of the child it also put the baby in the risk of hearing loss problem” (R4.7, 20 years)

The transmission of diseases from mother to child such as sexually transmitted infections (STI’s) and HIV/Aids were articulated by participants in a few GIs (6/17). Women across all age groups expressed their opinions that pregnant mothers with these conditions are more likely to give birth to babies that have HL.

“It is by STI’s, when having sex when pregnant while being infected, you will sometimes give birth to a child who have hearing problems” (O1.7, 18 years)

“It can be parents that are HIV positive have caused the child to be deaf” (L3.1, 25 years)

“When the mother comes to test her HIV status and found it negative, and never come back to repeat it in three months while her status changed to positive then the baby will be affected and have a problem of hearing loss.” (R3.1, 32 years)

Additionally, middle-aged women (7%) in a few GIs (6/17) said that pregnancy complication may lead to NHL

“It is a baby who is born pre-maturely, like 7 months. The body is not developed properly in other things” (O2.3, 26 years).

Regarding infant hearing loss, participants conveyed the causes of deafness as being rooted in environmental factors. In over half of the GIs (10/17), several middle-aged women (23%) emphasised that babies being brought up in noisy, dusty and unhygienic areas are more likely to acquire hearing loss. Their responses included:

“Too much noise may lead to hearing loss problem” (M1.7, 24 years)

“Hygiene may cause the loss of hearing, when you don’t clean the ear of a child s/he can get infection and put the child in the risk of hearing problem” (R2.2, 27 years)

In several GIs (9/17), views that ear infections developed by either dangerous objects being inserted in the ear or water entering the ear were expressed by some middle-aged women (14%) as follows:

“It can be an infection especially when the ears have a lot of discharge” (L3.4, 30 years)

“It is an infection which can block an eardrum” (O3.2, 24 years)

In a few GIs (5/17), various younger and older women (5%) viewed children that were subjected to physical and emotional abuse as more likely to have HL. This comprised of berating and hitting a child regularly.

“I think it is the baby that is always scorned and beaten by her/his parents” (M1.6, 18 years)

“When a child stays with non-biological parents who physically and emotionally abused her/him it will lead to deafness” (R1.2, 35 years)

Additionally, cultural factors were also acknowledged by several older and middle-aged women (8%) in a few GIs (5/17) as being the causes of hearing loss. The description was provided in the context of not adhering to traditional or ancestral rituals as well as spiritual, superstitions and bewitchment factors as expressed below:

“Sometimes a child will be affected if the family did not perform a new baby welcoming ritual. In our traditions, certain rituals need to be performed to welcome the child in the home” (L1.4, 28 years)

“Sometimes it can be a religious problem or sometimes the ancestors are punishing you or you maybe bewitched” (R4.3, 34 years)

“There are many causes that we all know, some are being told by our elders such as when an owl hoots on the roof of your house and baby is in the house, that baby will not hear again, unless the family perform specific rituals” (L1.5, 28 years)

Participants perspectives on causes of HL were expressed differently for newborns and older children, which led to the understanding of how women identify a child with HL.

#### Identification of deafness

We explored the possibility of participants ability to identify a child with HL in their own community. Participants said that a non-responsive and non-communicative child may be suspected of having a condition causing HL. In nearly all GI’s (15/17), women (11%) said that poor communication and no response in daily interactions was the highest indicator that a child is having a HL problem. These characteristics would be apparent in infants, toddlers and older children and was verbally stated by young and middle-aged women as follows:

“The child cannot play with others as s/he cannot recognise any sounds in their surroundings” (N4.3, 20 years)

“When you are giving instructions to your child, they might not respond unless you shout or get closer” (M1.2, 25 years)

In very few GIs (4/17), several middle-aged women (5%) said that a third-party member can inform the family of the likelihood of HL in a child. The third party may include teachers, children or a doctor. Such were their comments:

“The other children might pick it up when they are playing and report to parents” (L1.7, 28 years)

“Sometimes the teacher can tell you that a child has a problem and is not coping well at school. Then you can know that child had the hearing loss problem” (R4.1, 28 years)

Several middle-aged women (6%) in some GIs (4/17) said observable delays in child development can indicate a problem of HL:

“I think a parent can know because there will be difficulties and delay of talking.” (L2.1, 28 years)

“The child will not grow up like other children, s/he will delay in body growth.” (L3.4, 30 years)

The perspective that HL cannot be identified in a newborn was shared by younger and middle-aged women (2%) in very few GIs (3/17):

“It is not easy to identify a newborn baby that has a problem of hearing, maybe after two years” (R3.3, 20 years)

“If the child was born like that it will only be after three months you could notice as the sense of hearing is not developed yet” (L1.2, 26 years)

Some young women (8%), in very few GI’s (3/17), related soreness of ears as an indication of a HL problem and some also said they did not know how to identify a child with HL. As verbally expressed:

“If you touch their ears they cry and become aggressive about simple things” (M1.6, 18 years)

#### Detection and treatment

Participants were asked the possibility of detection of HL by health professionals in hospitals and possible treatments for CHL. In the majority of GIs (16/17), particularly middle-aged women (42%) said that HL can be detected by health professionals in a newborn, as they usually conduct several procedures after delivery and prior to being discharged. Their statements included:

“I think doctors can detect because they have skills and equipment to identify hearing loss” (N2.1, 21 years)

“Yes the doctor can detect that because they examine the baby before going home” (R4.1, 30 years).

There were several women (1%) in very few GIs (2/17) that were not aware, that the detection service for newborns could be provided by health professionals.

“I don’t think that doctors can diagnose if newborn baby has a problem of hearing loss, because at that stage the baby cannot hear anything” (R2.5, 21 years)

“It will be a little bit too soon for the doctors to see on a newborn, maybe when they are 6 weeks to 2 months” (O1.1, 24 years)

Nevertheless, in over half of GIs (13/17), some younger and middle-aged women (22%) responded that detection was impossible.

“No, I have three children and I have never heard any doctor saying that, so I do not believe it can be possible” (L3.3, 28 years)

“I don`t think the doctors can identify that, because sometimes they ask you ‘can the baby see, can the baby hear you’ I don`t think they can identify that problem” (R4.4, 19 years)

Furthermore, several younger and middle-aged women (12%) expressed their trust in the doctors and the health care facility as the only place where they can get treatment for a child with a HL condition.

“The doctors know what the treatments are. I will listen to what doctors says and I will do whatever they say” (O1.1, 24 years)

“The clinic and hospital is the only place that I can find the treatments of hearing loss” (R4.2, 18 years)

In very few GIs (4/17), some women mentioned that HL can be treated by traditional healers or with other remedies. Treatment done by traditional healers was described by a young woman as follows

“I will take to the *sangoma* (traditional doctor) because maybe the child has a problem of ancestors rather than wasting time to clinics” (R1.6, 19 years)

She further clarified about the treatment provided by traditional healers, as illustrated below:

“Using culture methods may be the best way to treat the hearing loss. There are some local herbs like (indlebelendlovu = ear of an elephant) from the traditional doctors they can cure the hearing loss problem” (R1.6, 19 years)

Other women explained that even natural home remedies are considered as treatment for HL:

“The breast milk of the mother can cure the hearing loss problem. … in my family my sister’s child had that problem of hearing and we were told by elders to put breastmilk and the ears became well” (R1.4, 21 years)

### Attitude

In exploring attitudes, two themes were identified comprised of beliefs and feelings.

#### Beliefs

Interactions of our everyday life shape what we think and believe. This theme was more about participants’ beliefs, opinions and thoughts about CHL and NHS. The gravity of a HL condition was believed to be the most problematic throughout one’s life. In the context of education and employment, in over half of the GIs (11/17), women believed that the child could experience challenges during his/her education that may result in difficulty in getting employment. Whereas, in a few GIs (6/17), younger and middle-aged women (5%) believed that due to lack of education the person will be dependent. As they commented:

“A child with hearing loss cannot cope at school…when it comes to her studies she cannot do well” (R1.4, 21 years)

“It will be hard for that person to get employed and will always be dependent on the family” (N1.1, 19 years)

“The child would not do well at school or may not get education and this would affect the family” (L3.10, 21 years)

Additionally, a lack of communication was believed by several middle-aged women (17%) of over half of the GIs (13/17) that the child would be vulnerable to many threatening situations, such as fire, vehicles on the road and rape. This viewpoint was conveyed as follows:

“The child would be in danger at all times–on the roads, near fires, so the family needs to look after all his life” (L2.7, 28 years)

“The child can easily be raped by someone because she will just give respect to older male on whatever they say” (R4.5, 23 years)

The lack of interaction with family members and community was believed by some younger and middle-aged women (10%) of a minority of GIs (4/17) to affect the child at personal level, leading to a feeling of depression and isolation. Women articulated this view in the following manner:

“When the child cannot hear, other children can tease him … which can drive the deaf child to psychological problem” (R2.3, 31 years)

“It is very difficult for the child with lack of communication s/he might have a feeling of isolation. S/he can even end up hurting herself such as committing suicide” (L3.2, 28 years)

“You become a joke in the community as people will just laugh at you” (N1.1, 19 years)

The feeling of isolation can also be due to the stigma and discrimination that can be experienced by the child within the community, as described by some women in a few GIs (3/17):

“It is a stigma in the community and families would always be afraid as the child would not be able to play with other children” (O2.4, 23 years)

“Some of families usually ignores the deaf child because s/he cannot communicate and discriminated against” (R3.3, 20 years)

Stereotypes, which can lead to discrimination, was also expressed by women as follows:

“Most of the deaf people are short tempered and it becomes difficult to communicate with them” (N2.5, 19 years)

Nonetheless, when participants were asked about their beliefs, in almost half of the GIs (7/17), women of all ages mentioned cultural factors to be associated with HL. These women felt that traditional healers were more capable of resolving hearing loss. They also believed HL to be a condition that can be solved spiritually.

“Those doctors at the clinic they do not help in those situations like that, I would rather go to find help in our cultural ways—traditional doctor (s*angoma*)” (R1.9, 18 years old)

“If there are no signs of improvement (at the clinic) I will go to church and pray to God” (L2.5, 29 years)

“As I am a Christian and I believe in prayers so I will think it’s a spirit and ask a church member to pray for me” (O2.3, 26 years)

#### Feelings

The ‘feeling’ theme arose from a question that asked participants’ how they would react, what they would do immediately and how would they feel if their child is identified with hearing loss. In all GIs, women expressed the feeling of being emotionally upset if informed that their child had a HL condition. These feelings of women varied between and within GIs. Participants in over two thirds of GIs (15/17), spoke about being emotionally upset in terms of crying, hurt, sad and unhappy. These following middle-aged women expressed the sense of helplessness, hopelessness and were inclined to self-pity as they see a bleak future for the child:

“I really don’t know what I do, but I will feel at a loss” (M2.4, 25 years)

“I will feel bad, because my child will not have a good future” (R4.5, 23 years)

Nonetheless, in over half of GIs (9/17), several younger and middle-aged women (8%) said that they would accept the situation and get whatever help was provided to them. The anticipated assistance comprised of seeing special doctors, sign language teachers and social workers. Some statements from women were as follows:

“I will be hurt and feel sad but I will become calm and take further steps to help my baby” (N1.5, 28 years)

“We need to ask the Department of Health to counsel us and help us to learn sign language, so that we can communicate with our babies” (O1.5, 20 years)

Other women, in very few GIs (2/17), said that even though they would be emotionally upset, they would call upon their family and relatives to make decisions.

“It will be painful, I will cry and talk to my family. They must decide what to do” (L3.10, 21 years)

“There will be nothing I can do, I will just cry and tell my family” (L2.4, 21 years)

### Practice

In terms of practice, three themes emerged: health seeking patterns, follow-up examinations and support systems.

#### Habitual health care practices/health seeking patterns

We all have certain habits that we follow when we are ill, which is determined by our circumstances and environment [22]. Women in this study explained their habits in seeking well-being during ill health. Most women, in all GIs, mentioned that their first choice of consultation was a professional health worker at the health facility. Most of the younger and middle-aged women (39%) visit a health facility whenever they are not feeling well:

“When I am not well, I normally seek medical help at the clinic” (N3.8, 18 years)

“I had always gone to the clinic, whenever I have a problem, as much as I can” (R4.5, 23 years)

The responses were similar even when they were asked where they would take a child if s/he is identified with HL. Some of the younger and middle-aged (23%) comments were:

“I will consult a doctor that can help with that problem rather than sit at home where I cannot find help” (R1.1, 18 years)

“I will take my child to the clinic for any treatment so that my child can be better” (M2.8, 24 years)

However, for a substantial number of women, in over half of the GIs (9/17), traditional healers were mentioned as the first point of consultation. This was expressed by younger and older women (7%) as follows:

“I normally go to the traditional healer, there are some local herbs that treat all everyday illnesses” (M1.1, 34 years)

“I usually go the traditional healer because most of the problems like hearing loss, eye problems and others are happening because of ancestors. So, traditional healers can tell me what I must do” (R1.9, 18 years)

In the context of their child being identified with HL, some of the middle-aged women (13%) stated:

“I will take the child to the traditional healer, maybe he will see the causes and find out what is wrong” (O2.10, 25 years)

“I will go to the traditional healer, maybe the child has been bewitched” (M1.4, 25 years)

The following middle-aged women, however, will visit traditional healers as a second option when they received unfavourable results from the clinic:

“I usually consult the doctor first, if I cannot get help, I consult the traditional healer” (R2.7, 27 years)

“I will go to the clinic first, then other means such as traditional healers or church” (O2.5, 25 years)

In a few GIs (5/17), some women reported their preferences to self-medication such as pharmacies and natural/local remedies rather than clinics. Some assertions from middle-aged and older women (6%) were as follows:

“Most of us do not go to the clinic because you stay long there due to long queues and not get much help. We prefer to go to pharmacy” (O3.5, 34 years)

“I go to clinic when it is necessary, I usually get medication from the chemist” (L1.4, 28 years)

“I usually pray and use natural remedies” (L3.3, 28 years)

Visiting the church when not feeling well was mentioned by some middle-aged women (7%) in a few GIs (4/17). Some of their comments include:

“I usually go to church and then at the clinic" (N2.4, 25 years)

“For me I usually get healed with prayers, I only come to the clinic for check-ups” (L2.5, 27 years)

“I normally go to church first and ask the pastor for a prayer after that I consult the doctor” (R3.4, 24 years)

Even when a child is identified with HL, their comments were similar such as:

“I will go to church and ask for a prayer because this will be a serious situation in my life” (R1.4, 21 years)

#### Follow-up examinations

This theme emerged after participants were asked about their willingness to comply with additional appointments arranged by the health professionals. In all GI’s, the majority of women were prepared to attend the appointments given. However, in most GIs, the extent of willingness varied between these women as some were ready to attend all scheduled appointments while others would not. Some of the comments of willingness to attend by middle-aged and older women (29%) were as follows:

“Every appointment that is scheduled, because I want my child to get help and be able to communicate with others” (M2.1, 33 years)

“As many times. I will follow all the instructions given by the doctor or nurses at the clinic” (N2.1, 21 years)

“I will honour all appointments” (L3.9, 27 years)

A small number of women (3%), from very few GI’s (3/17), mentioned attending limited appointments due to financial constraints, time and lack of trust of the devices used for check-up. These issues were expressed as follows:

“I will attend only two times in a year because I am not trusting those machines, maybe they can affect my baby ears” (R1.5, 29 years)

“Once a month, I have many other responsibilities and the day you come to the clinic it takes almost the whole day” (L1.2, 26 years)

“Quarterly when necessary, as transport cost money and there are many issues to resolve at home (L2.5, 27 years)

#### Support systems

As visitation to the clinic may be frequent when a child is identified with HL, having an understanding of support received from the family is important, as there would be minimal likelihood of failing to attend appointments. In over two thirds of the GIs (15/17), the majority of women asserted that they were more likely to go alone to seek treatment for their children. Younger and middle-aged women (42%) verbalised as follows:

“I go alone because there is no one who can go with me” (R2.4, 24 years)

“Women usually go alone with their babies at the clinic” (O1.7, 18 years)

“We normally go alone, I think everyone here agrees with me (the group nodded)” (N2.5, 19 years)

In almost a half of the GIs (7/17), several women said they preferred and received support from other members of the family and some from friends. This was expressed by younger and older women (14%) as follows:

“I prefer to be with someone like my sister because at any situation I need support. It is very sensitive with my immune system to get support from my family” (R1.7, 19 years)

“It will depend how sick the baby is, if very sick I go with relative, but if it is not serious I will go alone” (O3.3, 40 years)

Overall, the understanding of childhood hearing loss expressed by these mothers has generated a great deal of information and suggestions, which were subsequently constructed into nine themes as demonstrated above.

## Discussion

The overall purpose of this study was to explore maternal knowledge, attitude and practice towards CHL and NHS in a rural community to identify themes for the development of a KAP survey tool. The findings suggest various factors that need to be considered in designing such a tool as the understandings and meanings given to HL and NHS are complex, spreading across individual, family, community and cultural levels.

Contextually, gathering information of community KAP is essential to an ear health needs assessment to determine the potential acceptability of UNHS and to ensure effectiveness. Health needs assessments afford an understanding of the needs and integrates the results into service delivery [23]. The current study identified the diversity of knowledge associated with perception, causes and identification of deafness as well as detection and treatment. Although age related differences in overall perceptions were evident, they can be explained by a theoretical perspective of meaning, which refers “to the way self considers its past experiences” [20]. For example, the reported descriptions of CHL and its causes were greater with middle-aged women, followed by older women, with less from the younger women. This finding reveals that the middle-aged and older women responded according to their past experiences of either having a child or interactions with those with a child. The viewpoints of women reflected their personal experiences, everyday interactions and encounters with their world [20]. The younger women, on the other hand, lacked the experiences of being a mother and their interactions with others may have been limited. Additionally, despite the large percentage of single women, the above perspective of meaning, was unclear with regards to marital status as there was no difference of responses between married and single women.

These findings point towards constructing measures that can provide a description of women’s understanding of the HL and its intricacies. This includes constructing measures of causes; whether families can identify a child with HL; whether HL can be detected in a newborn and their comprehension of possible treatments. The potential construction of measures is similar in nature with the previous measurement tools that have primarily sought to understand families knowledge of infant HL and NHS [13,15,24–30]. These studies focussed on measuring participant’s views about the risk factors of infant HL and perspectives on NHS.

Disparities between the two are that the potential measures to be constructed from the findings of this study will be strongly influenced by the concepts shared by women regarding CHL and NHS, whilst the previous measures were largely influenced by biomedical science. Biomedical science pursues concepts within its culture, for example postnatal infection, ototoxic medication, in-utero infections, measles, jaundice, etc, included into previous measurement tools as risk factors of CHL [31–34]. Although the biomedical approach provides families’ needs in terms of knowledge or lack of it in relation to risk factors, it does not support the complex relationship between individuals and their settings, community and biology whereas this study set out to learn how mothers described their understanding of childhood ear health [35,36]. Accordingly, the apparent misconceptions about the causes of CHL from this study highlights the inadequate knowledge within the study group across all ages. This lack of knowledge will be factored in to the tool by constructing measures of all known and unknown causes of CHL. The developed tool could then capture the bigger picture of the community and eventually inform policy and practice and address the needs accordingly.

Other studies used tools that focused on measuring anxiety or satisfaction of families during or after the NHS processes by examining their emotions after the results of screening [14,26,27,37–39]. All these can be aggregated as attitude as the assessment involves a predisposition of participants to respond either positively or negatively towards NHS processes [40]. Generally, the descriptions of women’s beliefs about CHS and NHS demonstrated their evaluations of the study phenomena which has been influenced by existing events and experiences of everyday life, eventually shaping their beliefs. Conversely, women in this study indicated a continuum of emotional feelings during NHS process even when one’s child is identified with HL signposting to a potential construct measure. Although the emotional aspect of the findings sound like the measures of previous studies [12,14], the context is quite different particularly in relation to the timing of assessment. Previous studies’ measurements tools were mostly used during or after the screening process [14,24,38]. This study findings will allow for the potential tool to be used from the planning to the implementation phases of UNHS programme.

Additionally, findings also demonstrated themes which can allow us to understand the typical routines of women in seeking health, whether they are easily persuaded to attend further visits to health facilities as well as the existing support networks during child ill-health which will inform the practice domain.

The perspectives of parents in previous studies, in well-established UNHS in developed countries[12,41], compared to these findings, vary slightly in terms of individual experiences and context. The experiences of parents, in these countries, reflect well-resourced NHS services where the expectation of good outcomes are expected. In this study, the experiences of women exist where services are poor or non-existent. However, there are similarities between these countries and the study population with regard to the expected benefits of screening and the emotional impact on the mothers when a child is identified with HL [12,41].

Understanding mothers KAP in its context not only allowed us to identify themes that will enable us to develop a KAP survey tool, but also highlights the chances of making an impact since we met women within their own social cultural framework [36]. The guiding principle for the development of the tool is to contextualise the data into the knowledge, attitude and practice domains. Since the effectiveness of UNHSP depends on community acceptance of the services, the themes obtained would guide the development of the questionnaire. The study has identified four themes (perception, causes, identification of HL, detection and treatment) for the knowledge domain; three themes for the attitude domain and three themes for the practice domain. The themes identified indicated the strengths and limitations of the KAP which formed the basis for the development of questionnaire material.

### Limitations and implication to research

The limitations of this study include women being studied in isolation, where there are strong social structures and systems, and thus data collected may not be representative of the whole community. The KAP concepts obtained are embedded within their social and cultural aspects (religion, set of beliefs, traditions etc.) of everyday life which is dynamic and cannot be uncritically assumed to be the only truth. Hence, we might have failed to gather sufficient information on significant socio-cultural factors that could present challenges in the implementation of UNHS programmes. In addition, group interviews have a tendency of social desirability bias, although we tried to minimise this by using local facilitators and dialect during interviews [16,42], there is still a chance of the study being affected.

However, based on the themes obtained from this study, it would be possible to develop questions to capture data for the constructs of knowledge, attitude and practice. We believe that there is a potential to develop a KAP tool that would be broad enough to measure in detail all aspects of the three constructs.

## Conclusion

The study has established holistic data in terms of recognising the participants in the framework of the whole (where and how they expressed the phenomena meaningful) rather than assuming irrelevance (reduction or abstraction of data) to certain aspects of their explanations. Participants perspectives on CHL and NHS clearly demonstrates how identified themes were content sufficient in each KAP domain. The methodology in this study provides empirical information that directs us to the type of questions to be included in the survey tool. The questions will comprise of perception, causes, identification, treatment of CHL, as well as likelihood acceptance of NHS, their beliefs and feelings about early detection. It also clearly influences the responses to be incorporated in the tool and guides us to include the concepts of a community’s everyday language in relation to CHL and UNHS. Accordingly, it ought to be easier in terms of developing questions that are understood by future study participants in this community.

## Supporting information

S1 File Interview guide questions–Zulu version. (DOCX)

S2 File Interview guide questions–English version. (DOCX)

S3 File Themes–GI minimised data. (DOCX)
