# Supplementary material for: Mapping the content of mothers’ knowledge, attitude and practice towards universal newborn hearing screening for development of a KAP survey tool
Source: PLoS One. 2019 Feb 20;14(2):e0210764. doi: 10.1371/journal.pone.0210764 (PMC6382093; doi:10.1371/journal.pone.0210764)
Supplement: S1 File — (DOCX) [file pone.0210764.s001.docx]

**Groups Interviews – Guide Questions**

1. Yini oyaziyo ngokulahlekelwa ukuzwa?
2. Luthini uvo lwakho ngezingane ezizalwa zilahlekelwe ukuzwa? Yini ekwenza ucabange ngaleyondlela? Yini gqamayo emqondweni wakho ngalesisimo?
3. Kwenziwa yini umntwana agcine enenkinga yokungezwa ezindlebeni? Kudalwa yini ukungezwa? Uma ucabanga, yini imbangela?
4. Ngokombono wakho abanjani abantwana abangahle bebenenking yokulahlekelwa ukuzwa? Ubani ongalahlekelwa ukuzwa?
5. Ungazi kanjani ukuthi umntwana wakho unenkinga yokuzwa ezindlebeni
6. Ngokombono wakho kubucayi kangakanani ukulahlekelwa ukuzwa? Kungawuthinta kanjani umndeni nophakathi wakho ukulahlekelwa ukuzwa?
7. Uma ucabanga, kungenzeka yini odokotela esibhedlela bakhone ukubona inkinga yokungezwa ezindlebeni ezinganeni ezizelwe? Yebo noma Cha? Yini ucabange ukuthi bangakwazi noma bangekwazi?
8. Uma unganikwa ithuba lokuhlolelwa umntwana wakho amadlebe, ungavuma yini? Yini engakwenza uthathe lesosinqumo? Yiziphi izizathu ezikwenze wathatha lesosinqumo?
9. Ungenzenjani uma ungathola ukuthi umntwana wakho unenkinga engamenza agcine engezwa? isiphi isinqumo ongasithatha? Ungaphatheka kanjani? Sizathu sini esingenza uphatheke kanjalo?
10. Ungaphatheka kanjani uma umthwana wakho ungatholakala enenkinga yokungezwa ezindleni.
11. Uma untwana wakho engathola ithuba lokuxilongwa kabanzi, uzimisele ukumuletha kangakhi onyakeni? Ngobani?
12. Ungenzenjani uma uthola ukuthi umntwana wakho akezwa ezindlebeni?
13. Iluhi usizo ongalufuna uma ucabanga ukuthi umntwana wakho akezwa ezindlebeni?
14. Yini ocabanga ukuthi ingenziwa uma umntwana wakho enenkinga yokungezwa ezindlebeni?
15. Ucabanga ukuthi angalashwa kanjani?
16. Uma unganikwa amathuba okulashelwa umntwana wakho ezozwa kancono, unjalimulela yini? Ngobani?
17. Unokuyaphi uma ungaphathekile kahle, noma ukulashelwa ukugula?
18. Unokuya kangakhi ukuyofuna usizo, lwempilo emitholampilo noma esibhedlela?
19. Uma untwana uphathekile, abantu besifazane bavamise ukuya emitholampilo bebodwa noma baphelezelwa abomndeni?
20. Uma unengane engakhombisi ukiyizwa inkulumo yakho, noma enenkinga yokufunda, ungenzenjani?
21. Ulithole kuphi ulwazi ngokuhlolwa kwamadebe, kwezingane eziqeda kuzalwa?
