# Supplementary material for: Mapping the content of mothers’ knowledge, attitude and practice towards universal newborn hearing screening for development of a KAP survey tool
Source: PLoS One. 2019 Feb 20;14(2):e0210764. doi: 10.1371/journal.pone.0210764 (PMC6382093; doi:10.1371/journal.pone.0210764)
Supplement: S2 File — (DOCX) [file pone.0210764.s002.docx]

**Group Interviews – Guide questions**

1. What do you understand with hearing Loss or deafness?
2. What is your opinion about babies born with hearing loss? What makes you think that way? What stands out in your mind about the issue?
3. How does a baby get a condition of hearing loss? What are the causes? Why do you think it happens?
4. In your opinion, what kind of babies are more likely to have hearing loss? Who can have hearing loss?
5. How would you know that your child has a hearing loss condition?
6. In your opinion, how serious is a condition of hearing loss? What sort of impact does it have at family and community level?
7. Do you think that doctors at the hospital can identify hearing loss on a newborn baby?

- Why do you think they can do it and why not?

1. If you are offered a screening for your newborn baby, will you accept it? Why would you decide that way? What are the reasons for your decisions?
2. What would you do if your baby has been identified with a condition that might lead to hearing loss? What would you decide? How would you feel? Why would you feel that way?
3. How would you feel if your child has been detected with hearing loss?
4. If your child is offered more examination for hearing, how many times are you willing to come in a year? Why
5. If your child is found with hearing loss, what would you do?
6. What help would you seek, if you thought your child had a hearing problem?
7. What do you think can be done when a child has a hearing loss?
8. What do you think are the treatments for hearing loss?
9. If provided with opportunities for treatment so that your child can hear better, would you accept it? Why and why not?
10. Where do you usually go if you are not well, or for treatment of general health problem?
11. How often do you generally seek health at the clinic or hospital?
12. When a child is not well, do women usually go to a health care facility alone or are they accompanied by their relatives?
13. If you had a child who is not responding to your communications or difficulty in learning what would you do?
14. What has been your primary source of information about health issues?
