# Supplementary material for: Mapping the content of mothers’ knowledge, attitude and practice towards universal newborn hearing screening for development of a KAP survey tool
Source: PLoS One. 2019 Feb 20;14(2):e0210764. doi: 10.1371/journal.pone.0210764 (PMC6382093; doi:10.1371/journal.pone.0210764)
Supplement: S3 File — (DOCX) [file pone.0210764.s003.docx]

**Minimised data - themes**

Theme: Perceptions of deafness

| Aged: 18 - 20 | Extracts |
| --- | --- |
|  | "it is when a person cannot hear anything and he cannot communicate with others” |
|  | “Sounds waves does not enter in the ear and the vibrations are not good, it is a blockage in an ear.” |
|  | “If the child is among the community that does not understand him/her the chances of discrimination are likely to happen” |
|  |  |
| Aged: 21 - 30 | “If a person cannot respond to any noise in their surroundings that means the person is deaf” |
|  | “Babies born with hearing loss needs to get special treatment before it gets worse. If delayed it can damage baby’s eyes and the baby will be incapacitated.” |
|  | "I will need to take her to the clinic and get help because it is not good at all to have a baby with a hearing loss problem" |
|  | "The government need to do something like awareness about that problem of hearing problem maybe it will work and save those children that have the problem. |
|  | "it is a problem with ears" |
|  | “With my understanding I think it is a problem with an eardrum” |
|  | “I think it’s a nerve in the ear that is not right” |
|  | "As mothers, there is a need for some professional help so that they counsel us and tells us which steps we can take from now on so that our babies can get better". |
|  | " I think the babies need to get immunization it might help" |
|  | " I have no knowledge, but I have seen deaf people" |
|  | " No, knowledge" |
|  | " I think it is a person who cannot hear and sometimes not able to speak " |
|  | "Ahh!!! Babies born with hearing loss? I don’t know" |
|  | " It may be a disease which cause a person not to hear properly " |
|  | “I have never heard of a baby being born deaf” |
|  | “It is not possible for a baby to be born deaf. I want to know, does this happen during pregnancy and what could cause it?” |
|  | “It cannot be the baby is still too young for hearing anything” |
|  | “It will be painful for the mother but the baby has no fault and will need help.” |
|  | “Maybe it would be necessary to check the parents if they are the cause of the problem” |
|  | “There is nothing to think about it is God’s will, I will not do anything” |
|  | “It is a person that have hearing problems" |
|  | “That child will have problems at home with other children and when s/he start schooling. The challenges will be hearing other children and at school.” |
|  | “It is when somebody doesn’t hear when you talk to them.” |
|  | “You must immediately took your child to the clinic” |
|  | “Parents should take precautions at early stage when they suspect that the child’s hearing is not good and take actions in that matter to find out what’s wrong. Because when the child start schooling she must be able to hear clearly what the teacher is saying. Sometimes as parents we usually take our kids to normal schools without noticing that the child need special attention.” |
|  |  |
|  |  |
| Aged: 31 - 40 | “I think it is when you cannot hear any noise made around you” |
|  | "When a baby born with hearing loss, it is a problem because no one will handle that matter. A mother will have to find a special school for communication between mother and child a mother have to learn a sign language". |
|  | “I think the problem is with eardrums of the baby that might cause the problem in hearing” |
|  | “I think it is the difficulty in hearing” |
|  | “I think the baby must get the help quickly so that the baby can get treatment.” |
|  | “It is when a person has difficulty in communication with other people because s/he cannot hear **“** |

Theme: Causes of Deafness

| Aged: 18 -20 | Extracts |
| --- | --- |
|  | "Those babies whom their mothers did not get healthy food while pregnant" |
|  | "I think it might happen if there is another family member who is deaf and that can be passed on to the unborn babies too". |
|  | "It is by STI’s, when you are having sex when pregnant you will sometimes give birth to a child who have hearing problems" |
|  | "It is caused by an infection which can block the eardrum". |
|  | “When you bath the baby we need to be carefully because when the water get into an ears of the baby it can affect and lead to hearing loss problem.” |
|  | "It is a hereditary disease, for example, when one member of the family has a hearing problem, then a child might be affected". |
|  | "when the mothers are used to not coming to the clinic for immunization of the child it also put the baby in the risk of hearing loss problem" |
|  | “I think is the baby that is always scorned and beaten by her parents” |
|  |  |
| Aged: 21 - 30 | "When the pregnant mother uses drugs or is taking alcohol that will affect the baby and become deaf". |
|  | "Those babies whom their mother started the clinic late during their pregnancy” |
|  | "Not eating healthy food". |
|  | "The children whose mother’s drink alcohol and smoke during pregnancy". |
|  | "It is a baby who is born pre-maturely like 7months the body is not developed properly in other things". |
|  | "Sometimes kids play with dangerous things like matches in their ears that can lead to hearing loss problem". |
|  | "I think it can be caused by mothers starting clinic very late" |
|  | "Hygiene may cause the loss of hearing, when you don’t clean the ear of a child s/he can get infection and put the child in the risk of hearing problem" |
|  | "it is the type of feeding that can affect the baby such as at birth you feed her/him with the formula it might affect the baby and have hearing loss". |
|  | "Sometimes kids play with dangerous things like matches in their ears that can lead to hearing loss problem". |
|  | "When a child is abused most of the times, especially by hitting her on the head she will be affected ". |
|  | " It is painful to think about that, I think it is caused by too much noise but I will seek help" |
|  | "My husband told me that drinking alcohol and smoking when pregnant causes many diseases such as child blindness and many others, so maybe even this one." |
|  | "I agree with that maybe it is genes, if someone in the family is deaf than children can be born deaf as well." |
|  | "Sometimes a child will be affected if the family did not perform a new baby welcoming rituals. In our traditions certain rituals need to be performed to welcome the child in the home." |
|  | "I agree with that especially when you go home. But also there are many causes that we all know being told by our elders such as when an owl hoots on the roof of your house and baby is in the house, that baby will not hear again, unless the family perform specific rituals" |
|  | "I don’t know what causes a child to be deaf, so it is difficult to explain the causes" |
|  | “Maybe we can just look at it as God created them like that” |
|  | "It can be that the mother did not get immunization during pregnancy." |
|  | " It can be hereditary, as there are some problem which are passed on to children" |
|  | “I think God created the child that way and there is no need to look for causes” |
|  | “It can be an infection especially when the ears have a lot of discharge” |
|  | “It can be parents that are HIV positive have caused the child to be deaf” |
|  | "I am not sure maybe even mothers who had STI during pregnancy". |
|  | “Sometimes certain injuries happens during delivery that can cause the problem” |
|  | “Don’t know, only God knows” |
|  | “I think it may be caused by pregnant mother taking too much alcohol. I have a relative who was doing that when pregnant and the child was born with a lot of problems with ears that were always discharging.” |
|  | “It might happened that the child has been born with the hearing problem due to mothers’ behaviour during pregnancy. Such as drinking alcohol or being physically abused by the husband” |
|  | “It can be anyone” |
|  |  |
| Aged: 31-40 | "It is for mothers who ... did not go for immunization". |
|  | "When a child stays with the non-biological parents who physically and emotionally abused her/him which leads to deafness and hearing loss". |
|  | "The babies that a brought up in louder sounds like radios are in danger of hearing loss problem or deafness". |
|  | "When the mother is coming to test her HIV status and found it negative, and never come back to repeat it in three months while her status changed to positive then the baby will be affected and have a problem of hearing loss. |
|  | "Sometimes it can be a religious problem or sometimes the ancestors are punishing you or you maybe bewitched" |
|  | "When a child stays with the non-biological parents who physically and emotionally abused her/him which leads to deafness or hearing loss". |
|  | “Playing outdoors on windy weather can cause hearing loss to a baby” |
|  | “Too much noise may lead to hearing loss problem” |
|  | “Maybe it is genetic. It is a condition that can be passed on from one generation to another” |
|  | “A baby will have a wound that bleeds inside the ear and this could cause hearing loss.” |
|  | |

Theme: Identification of Deafness

| Aged: 18 – 20 | Extracts |
| --- | --- |
|  | "It is not easy to identify the newborn baby that has a problem of hearing, maybe after two years" |
|  | "The child cannot play with others as s/he cannot recognise any sounds in their surroundings". |
|  | At an early stage of pregnancy there’s a process done by doctors, they give an injection to take immunity fluids and check for any problems so that they can help my baby. |
|  | “If you touch their ears they don’t cry and also they become aggressive about simple things**”** |
|  | "If you talk to the child and s/he is not responding you can know that the child has a problem of hearing loss". |
|  |  |
| Aged: 21 - 30 | "Sometimes the teacher can tell you that child had a problem and is not coping well at school you can know that child had the hearing loss problem". |
|  | "your child doesn’t like to play with others then you will know that child has a hearing loss problem" |
|  | " If the child was born like that it will only be after three months you could notice as the sense of hearing is not developed yet " |
|  | "The child would not be able to understand what others are saying and just being confused with whatever asked to do" |
|  | “The child would not respond to verbal commands” |
|  | “The other children might pick it up when they are playing and report to parents” |
|  | "It is difficult, but maybe when he grows up other children will tell the parent." |
|  | "Yes, you may get a report from the teachers at school ". |
|  | " I don’t know" |
|  | “The doctors will know" |
|  | “I think a parent can know because there will be difficulties and delay of talking.” |
|  | “Yes, the child will not grow normally, like even to say the first words will come very late.” |
|  | “The child will not be responding to any sounds” |
|  | “The child will not respond even when I play with the child.” |
|  | “The child will not grow up like other children, s/he will delay in every body growth.” |
|  | “The child will be not perform well at school” |
|  | “When you are giving instruction to your baby, the baby might not respond unless you shout or come closer to.” |
|  | “They child will not respond when you talk to him/her” |
|  |  |
| Aged: 31 - 40 | I think there’s a machine at the hospital that check the baby first and do the test (like for an example, if the test comes with a green line they said your baby are ok. ORANGE be aware there is a condition not right. RED your baby are not hear clear. |
|  | “The child would not like to play with others as it will be difficult to communicate” |
|  | “If you speak to her/him they do not respond.” |
|  | “Even if there is a huge noise they are not able to hear.” |
|  |  |

Theme: Detection and Treatment

| Aged: 18 - 20 | Extracts |
| --- | --- |
|  | "I don`t think the doctors can identify that, because sometimes they ask you ‘can the baby see, can the baby hear you’ I don`t think they can identify that problem.” |
|  | "Using culture methods maybe the best way to treat the hearing loss there are some local herbs (indlebelendlovu=ear of an elephant) from the traditional doctors they can cure the hearing loss problem. |
|  | "The clinic and hospital is the only place that I can find the treatments of hearing loss". |
|  | "go the clinic so that the can arrange for my baby to be transferred to hospital for more counselling" |
|  | "I will take to the Isangoma (traditional doctors) because maybe the child has a problem of ancestors rather than wasting time to clinics". |
|  | “I will take my child to the clinic” |
|  |  |
| Aged: 21 - 30 | "I will try both traditional doctor as well doctors from hospital because I don’t know where I will find help because this hearing loss problem will be a serious issue". |
|  | "I will take her to the clinic or to a doctor who specializes with ears because I do not want my baby found in that situation" |
|  | "I don’t think that doctors can diagnose a newborn baby with a problem of hearing loss, because at that stage the baby cannot hear anything" |
|  | "Yes the doctor can detect that because they examine the baby before going home". |
|  | " it will be a little bit too soon for the doctors to see hearing loss on a newborn, maybe when they have 6 weeks to 2 months" |
|  | "No, they won’t identify because on my understanding the sense of hearing will develop when a child gets older". |
|  | "The breast milk of the mother can cure the hearing loss problem. For example, in my family my sisters child had that problem of hearing and we were told by elders to put breastmilk and the ears became well". |
|  | "The doctors know what the treatments are. I will listen to what doctors says and I will do whatever they say" |
|  | "Audiologist will tell me what treatment my baby can get". |
|  | "Yes, doctors can do, I think so because they have skills and equipment to identify hearing loss". |
|  | "Yes doctors can, as they are examining the babies before being discharged" |
|  | “I think Doctors can even detect it before birth” |
|  | “They can detect it” |
|  | "I will get hearing aids" |
|  | "Maybe doctors can do an operation to make the child hear again" |
|  | “I would say that doctors can take other new measures that I don’t know” |
|  | "I will take her to the clinic to get artificial hearing by inserting new things in the ear" |
|  | "If there is no treatment, I will send to special schools" |
|  | “Yes, they can and maybe they can operate on the baby” |
|  | “Yes, it may be difficult but with the new technology nowadays anything is possible” |
|  | “Yes, but I don’t know how only God can” |
|  | “That is true no one is above God, so I say no” |
|  | “We will go to the clinic and if the treatment failed and agreed that the child is deaf then we will take her to deaf schools to learn sign language” |
|  | “As said earlier, I will take the child to the doctor and the church for prayers” |
|  | “I will call the family and explain the problem of the child maybe they will help to take the child to the doctor” |
|  | “I will find out with Social workers whether they can help the child” |
|  | " Yes maybe they can through amniocentesis" |
|  | "No, not to a newborn baby, maybe after a few months". |
|  | “No, I have three children and I have never heard any doctor saying that, so I do not believe it can be possible” |
|  | “No, I agree with you most of us usually deliver at the clinics where there are no doctors only nurses” |
|  | “I will accept the child as my gift and will not do anything” |
|  | “I will first find help from the clinic then look for the special school” |
|  | “I will talk to my family and find help to take the child to special school” |
|  | "I will take the child to the clinic for advice" |
|  | "Doctors can give something if they know the cause " |
|  | "I don’t know" |
|  | “Maybe an operation and hearing aid.” |
|  | “Yes they can diagnose the child.” |
|  | “None, I don’t think there is any treatment for deafness.” |
|  | “If I found out that my child is not deaf and only have a condition of hearing loss I will be happy for my child to use hearing aid if the child is deaf I then go for sign language” |
|  |  |
| Aged: 31 - 40 | "I will take him to doctors and do whatever they say". |
|  | “Yes, because they have devices that can detect any ear problem” |
|  | “Maybe yes as they are taught about the babies and with the new machines” |
|  | “Yes, so that the child can get full medical help urgently” |
|  | "If it happened that the child is deaf I think the sign language will be the solution or if it is a hearing loss hearing aid will be ideal" |
|  | “Yes, Because they are trained to do so” |
|  |  |

Theme: Beliefs about deafness/hearing loss

| Aged: 18 - 20 | Extracts |
| --- | --- |
|  | "It is serious but some of families usually ignores the deaf child because s/he cannot communicate and discriminate him. The child would start to do things that are not good in life like use of drugs" |
|  | "I will accept it (screening) because maybe I will get help earlier and I might prepare to learn sign language so that I can communicate with my child". |
|  | "I will not accept it (screening) because those doctors at the clinic they do not help in those situations like that, I would rather go to find help in our cultural ways - traditional doctor - Sangoma". |
|  | "I will seek help from the Department of Social Development to come at home and to check and do assessment" |
|  | "It is serious, as the deaf person will not be able to find a job and the family will have to support him." |
|  | "It is serious because you can think the person is difficult as deaf people can be angry for nothing" |
|  | "Yes, I will accept, because I need a better life for my child, and I also need my baby to grow healthy". |
|  | “Most of the deaf people are short tempered and it becomes difficult to communicate with them” |
|  | “It will be hard for that person to get employed and will always be dependent on the family” |
|  | "I will visit the church" |
|  | “You become a joke in the community as people will just laugh at you” |
|  | “It will be difficult for the family and community to communicate to a child who has a hearing loss” |
|  |  |
| Aged: 21 - 30 | "A child with hearing loss cannot cope at school because other learners will always gossip about her and even when it comes to her studies she cannot do well". |
|  | "It is serious. The child can be depressed because s/he cannot communicate with others at home and also cannot cope well at school because of hearing loss problem" |
|  | "The child can be easily raped by someone because she can just give respect to older male but not knowing what he said to her". |
|  | "I will accept but I must talk to my family first in my home we use culture, I mean sangoma (traditional doctor) too much maybe they will not accept it" |
|  | "I will consult the doctor as quickly as possible because it is really serious to have a child that had a hearing problem" |
|  | "The cultural practices are the best, the traditional doctor can help me". |
|  | "It will affect the family because at home there is a person who don’t speak and another have a hearing loss, so we all have to speak with sign language. That is where my family has been affected" |
|  | "It is a stigma in the community and families would always have fear as their child would not be able to play with other children" |
|  | "It is difficult especially when he walks at the street and does not hear the sound of a hooting car". |
|  | “As I am a Christian and I believe in prayers so I will think it’s a spirit and ask a church member to pray for me” |
|  | "If I go to the doctor’s and if don’t get help, we are black people and we believe traditional healer’s, I will go there for seeking help and also with a Zulu religion". |
|  | "I will seek Audiology doctors to tell me exactly what is affected, and tell me what to do and I will follow those steps". |
|  | "It is serious because they are short tempered" |
|  | "If I can’t get help from the clinic I will consult traditional healers" |
|  | "If I can’t get help from the clinic I will consult traditional healers" |
|  | "It is very serious, it will be very painful for the mother and family. The child could get injured easily and s/he is unaware of her/his surroundings" |
|  | "It is serious. It is costly to educate these people. The government must intervene." |
|  | "It is serious. In our community it will be bad as we have no facilities for their education." |
|  | "I will go the doctor for hearing aids. It is hurting but I must live with it. " |
|  | "I will take the child to the professionals ". |
|  | "I will go to the clinic or doctor for help" |
|  | "I will go to the clinic, but if my child does not get better I will go to the traditional healer " |
|  | "I will ask the school health team to assist" |
|  | "I will look for the special school". |
|  | "It is a big problem because there would be no proper communication between the child and the family and community". |
|  | “The child will be a slow learner and will have difficulty to get employment” |
|  | “There are very few people who know the sign language thus the child will not have a good flow of communication” |
|  | “The family as well as the community will experience problems of communication” |
|  | “It is serious, as the child would be in danger at all the times – on the roads, near fires, so the family needs to look after him all his life” |
|  | “As there will be difficulty in communication with the child, s/he may feel neglected or unloved this might lead to suicide.” |
|  | “I will find people who knows the doctors that can help me” |
|  | "I would ask if there could be any help if not I will accept things as they are I cannot change the will of God" |
|  | "I will send the child to school to learn sign language " |
|  | " Take the child to professionals that will examine and help in solving the problem" |
|  | " I will take to the clinic and ask to see a doctor " |
|  | " It is very problematic as the person cannot be helped by many people even when in danger, there will be no communication" |
|  | “It is difficult to bring up a deaf child like others due to communication problem. The child needs support all the time and you don’t know what s/he wants.” |
|  | “It will be difficult but we must accept the child as s/he is as it is Gods will” |
|  | “It will be difficult even when they grow up to socialise or participate in family or community events” |
|  | “It is very difficult for the person as the communication problem may left her out and the feeling of isolation. She can even end up hurting herself such as committing suicide” |
|  | “It will be a problem for the child as she would not do well at school or may not get education and this would affect the family” |
|  | “It can be a big problem for the family as they cannot leave alone in all their lives the family has to support them.” |
|  | “I will take the child to the clinic for help” |
|  | “I will accept the child as s/he is” |
|  | “I am not sure” |
|  | "I will live with it it’s a gift" |
|  | "Take to the clinic" |
|  | "Take to special schools" |
|  | “I will go to Social workers to ask for help” |
|  | “I will go to a traditional healer, maybe the child has been bewitched! |
|  | “It is serious, since one cannot hear what is happening in his surrounding, other people can take advantage of him in different situations. Like people may gossip about the person” |
|  | “Even when walking on the road you may not hear when cars behind you are moving towards you.” |
|  | “It does have impact to the family because a deaf person would always require support from the family members. For example, if a person wants to go somewhere then a family member would accompany the person wherever he goes in order to assist him” |
|  | “I would seek help immediately before it get worse or I will send my child to special school for sign language” |
|  | “I will take my child to the doctors so that the child can be examined” |
|  | “I will go to a Sangoma” |
|  |  |
| Aged: 31 - 40 | "Yes I will (accept screening) because I want my baby to have a good healthy life, and it is important to know any problem my kid has in life and to treat it early" |
|  | "to consult the clinic because the clinic is where I can get help" |
|  | "It will affect my family and community because now there is a case where people can rape our child and the child cannot communicate what has happened to him/her". |
|  | " Yes, nobody wants to stay with a baby that have a condition of hearing loss, no one will be happy with that problem" |
|  | "I really don’t want to think about it" |
|  | “It is serious but the family must contribute on learning sign language to support the child.” |
|  | “It is serious because the child becomes discriminated against because of the condition.” |
|  | “The condition is very serious because the child will not be able to hear or understand what is going on.” |
|  | “I will ask the sister/doctor who was testing the baby what will happen next.” |
|  | “I will take my baby to all the treatments suggested by the clinic” |

Theme: Feelings

| Aged: 18 - 20 | Extracts |
| --- | --- |
|  | "I will feel very bad because my child is my first priority, is my life, my happiness and my responsibility". |
|  | "We need to ask the Department of Health to counsel us and try to teach us as mothers a sign language, so that we can communicate with our babies" |
| Aged: 21 - 30 | "I will feel bad because my child would not be able to cope well at school" |
|  | "I will feel bad, because my child will not have a good future". |
|  | "I will be sad but will to take all means for my baby and learn the sign language" |
|  | "I will not be happy but I have to accept it, so that my baby can accept the condition s/he has". |
|  | "I will be hurt and feel sad but I will become calm and take further steps to help my baby to survive.” |
|  | "It will be very painful and a feeling of sadness" |
|  | "It will be very painful" |
|  | "Yes very emotional but I will seek for help wherever I can get it" |
|  | "It is difficult to express how I would feel" |
|  | "I will accept it though it is painful" |
|  | "It will be sad and painful especially when the child has been taught sign language at school and we at home do not know how to communicate with him" |
|  | "There will be nothing I can do I will just cry and tell my family" |
|  | "I will be terrified, cry a lot and ask for my mother’s advice" |
|  | "I will feel bad, accept the circumstances as they are" |
|  | “It will be painful, I will cry and talk to my family they must decide what to do" |
|  | “I will be frustrated but take the child to clinic for more help” |
|  | "I will feel really bad but I will take child to clinic, I don’t want to think about it" |
|  | “I will be sad and helpless but will still go to the clinic for any hope” |
|  | “I will feel bad I will go the clinic” |
|  | “I cannot even think about that, I will just call my family crying” |
|  | “Helpless.” |
|  | I will feel bad because my child will not have a good future” |
|  | “I will be sad.” |
|  |  |
| Aged: 31 - 40 | "I will be hurtful and I will cry". |
|  | “I will be sad” |
|  | “I will feel so sad but will take my child to a doctor I will also seek advice from counsellors on how to learn the way of communicating with sign language” |
|  |  |

Theme: Health Seeking Patterns

| Aged: 18 - 20 | Extracts |
| --- | --- |
|  | "I usually go to the traditional doctors because some of those problem like hearing loss, eye problem and others is because of ancestors so the traditional doctor can tell me what I must do". |
|  | "We need the doctors to examine the child at the clinic there is no other way we can do as mothers." |
|  | "I will consult a doctor that can help with that problem rather than sit at home where I cannot find help". |
|  | "To the pharmacy/chemist". |
|  | " I will go to the clinic for further investigation" |
|  | "I usually go to the church and then at the clinic." |
|  | "Doctors will take the baby for check-up." |
|  | "When I am not well I normally seek medical help at the clinic." |
|  | “When not feeling well” |
|  |  |
|  |  |
| Aged: 21 - 30 | "I usually consult the doctor first if I cannot get help I consult the traditional doctor. |
|  | "I normally use traditional healers when I have the problem in my general health problem because sometimes you cannot get help in the clinic". |
|  | "I normally go to church first and ask the pastor for a prayer and after that I consult the clinic or the doctor". |
|  | "I will go to the chemist and tell them what my problem and then they will help me". |
|  | "I used to go many times when I had a problem up until reach the stage that I can get help". |
|  | "I go so many times until I get cured to that problem". |
|  | "I had always gone to the clinic, whenever I have a problem, I had I always come the clinic". |
|  | "I used to go many times when I had a problem up until reach the stage that I can get help". |
|  | "I will go to find help from those school who specialize about those children had the problem like that". |
|  | "I will go to the church and look for a prayer because this will be the serious condition in my life". |
|  | "I will consult social workers to try to get help". |
|  | "To the traditional healer, so that I can get IZIMBIZA (local herbs that helps cleanse the blood)". |
|  | "I will go to church". |
|  | "As I am a Christian and believe in prayers so I will think it’s a spirit and ask a church member’s to pray for me". |
|  | "I will take the child to the traditional healer, maybe they will see the causes and find out what is wrong" |
|  | "I will go once or twice in a month when I am sick". |
|  | "I will go first to the clinic, then other means such as traditional healers or church". |
|  | "I will start with the clinic then traditional healers" |
|  | "Whenever I am sick" |
|  | "I will take the child to the clinic." |
|  | "To the clinic to see the doctor" |
|  | "Clinic" |
|  | "Seldom, when not well" |
|  | "When it is necessary" |
|  | "When I feel it is necessary, I usually get medication from the chemist " |
|  | “I usually pray to God then the clinic” |
|  | “Clinic then they can send me to doctor” |
|  | “To the chemist” |
|  | “At the time when I am feeling sick” |
|  | “For me I usually get healed with prayers I only come to the clinic for check- ups” |
|  | “Usually to the Chemist” |
|  | "Take the child to professionals that will examine and help in solving the problem" |
|  | "I usually go to the clinic" |
|  | “ I usually pray and use natural remedies I also go to the clinic" |
|  | “I only go to the clinic when serious but usually to the chemist” |
|  | “To the chemist” |
|  | “Take the child to the doctor” |
|  | “When not well” |
|  | “Only when I am pregnant” |
|  | “Only when I am seriously ill” |
|  | “Anytime when I am not well” |
|  | “I will go to a traditional healer, maybe the child has been bewitched” |
|  | “Yes I will accept the treatment so that my child can be better.” |
|  | “Yes, if there are any treatments I will, so that my child can be better.” |
|  | “Yes, I want my child to be same as others and feel at home” |
|  | “Clinic” |
|  | “I usually go to Sangoma (Traditional healer) to get herbal medicine” |
|  | “Once a month for check-ups” |
|  |  |
| Aged: 31 - 40 | "I go to a doctor to get help when I have a problem because they is no other place I trust". |
|  | "I will take the child to the doctors that specialize with hearing loss problem" |
|  | "I will just go to the clinic for two to four times and if I there is no help I just leave and go to traditional doctors" |
|  | "To the clinic" |
|  | "I think the doctors will do an operation maybe there is a blockage". |
|  | "Most of us don’t go to the clinic because you will stay long and not get help, there are long queues we prefer to go to the pharmacy" |
|  | "I will go to the clinic and maybe they will refer me at the hospital for sign language and reading" |
|  | "I usually go the clinic to seek help and treatment." |
|  | “I usually go to the traditional healer, there are some local herbs that treat all everyday illnesses. If serious to the clinic” |
|  | “At the clinic” |
|  | “When I am sick” |
|  |  |
|  |  |

Theme: Follow-up examination

| Aged: 18 - 20 | Extracts |
| --- | --- |
|  | "I will go every week, so that they will check for any other things that comes up". |
|  |  |
|  |  |
| Aged: 21 - 30 | "I will come as many times because I don’t want my baby to be in danger of hearing loss." |
|  | "I will attend only two times in a year because I am not trusting those machine maybe they can affect ears of my baby" |
|  | "As long as I can find help I will come many times." |
|  | "I think as long as they ask me again I will come". |
|  | "A thousand times as long as my baby can get help" |
|  | "Several times almost the whole year, so that they can give my child more examination". |
|  | "I will come each and every month so that they will help my baby early" |
|  | "As many times. I will follow all the instructions given by the doctor or nurses at the clinic ". |
|  | "As many times, all the appointments made for my baby" |
|  | "Once a month, I have many other responsibilities and the day you come to the clinic it takes almost the whole day" |
|  | "Every day if that is what I am supposed to do" |
|  | "Quarterly when necessary as transport cost money and there are many issues to resolve at home" |
|  | "I will attend every appointment" |
|  | "I don’t know as long as they say I must bring the child and I have the money for treatment" |
|  | "I don’t know as long as they say I must bring the child and have money for treatment " |
|  | " All times according to the appointments" |
|  | " As often as I am supposed to" |
|  | " I will honour all appointments" |
|  | “I will take my child on every appointment” |
|  | “Yes, every month if they asked me to.” |
|  |  |
|  |  |
| Aged: 31 - 40 | "as many times just to be updated of the ears of my child if they are still good or not" |
|  | “On all appointments that will be given to me for check-up. |
|  | “Every appointment that is scheduled, Because I want my child to get help and be able to communicate with others” |
|  |  |

**Theme: Support Systems**

| Aged: 18 - 20 | Extracts |
| --- | --- |
|  | "I prefer to be with someone like my sister because at any situation I need support because it is very sensitive to my immune system. When I get the support especially from my family I have hope". |
|  | "Women usually go alone with their babies at health care facilities" |
|  | "We normally go alone, I think everyone here agrees with me" (OTHERS NODDED). |
|  |  |
| Aged: 21 - 30 | "It depends on the situation, if the baby is sick and not strong I will need the support from my family but when it is just normal sickness like flu I go alone" |
|  | “I go alone because there is no one who can go with me” |
|  | "I always go with the father of my baby". |
|  | " With a friend” |
|  | " With my mother" |
|  | " With my sister " |
|  | " My mother or sister" |
|  | " My husband" |
|  | " My mother or father" |
|  | "I always go with the father of my baby". |
|  | " Alone" |
|  | " Alone" |
|  | " Alone" |
|  |  |
| Aged: 31 - 40 | "I need a company especially my relatives to support me because it is not easy to accept any condition that have been seen to a child, when comes to a child I will need support". |
|  | “My family supports me and come with me to the clinic when the child is not well”. |
|  | "Go alone with my child". |
|  | "It will depend how sick the baby is, if very sick I go with relative, but if it not serious I will go alone". |
|  | " Alone" |
|  | " Alone" |
|  | " Alone" |
|  | " Alone" |
|  | " Alone" |
|  |  |
